# Academic achievement and socio-demographic differences in ADHD diagnosis: a population-based study of Swedish compulsory school pupils

**DOI:** 10.1186/s13034-026-01176-4

**Published:** 2026-09-09

**Authors:** Björn Högberg

**Affiliations:** 1 Department of Social Work, Umeå University, Umeå, SE-901 87 Sweden; 2 Centre for Demographic and Ageing Research, Umeå University, Umeå, Sweden

**Keywords:** ADHD, Gender, Socio-economic status, Immigration, School

## Abstract

**Background:**

Attention-deficit/hyperactivity disorder (ADHD) is more commonly diagnosed among boys, children with lower socio-economic status (SES), and native-born compared with immigrant children. ADHD can strongly impair academic achievement, and impaired academic functioning can also trigger diagnostic referral. The aim of this paper was to investigate how socio-demographic differences in ADHD diagnosis change when differences in academic achievement are taken into account.

**Methods:**

Population-based cross-sectional study using linked national registers, comprising 296,380 pupils graduating from Swedish compulsory school (grade 9, age 16) between 2015 and 2017. ADHD was defined using specialist diagnoses and dispensed ADHD medication. Academic achievement was measured by compulsory school-leaving grades. Logistic regression models were estimated with and without adjustment for achievement, and estimates were transformed into marginal effects and predicted probabilities.

**Results:**

Before adjustment for achievement, boys had a higher probability of ADHD diagnosis than girls, pupils with tertiary-educated parents had a lower probability than those with compulsory-educated parents, and immigrant pupils had substantially lower probability than native pupils. Adjustment for achievement reduced the gender gap by approximately two thirds, reversed the SES gradient such that pupils with tertiary-educated parents had the highest probability, and slightly widened the native-immigrant gap. When accounting for differential timing of diagnosis, the gender gap even reversed, with a higher probability among girls than among boys.

**Conclusions:**

The findings underscore the need to consider academic achievement when interpreting socio-demographic patterns in ADHD diagnosis, and suggest that some groups may face barriers to diagnosis even when their level of academic impairment is comparable to that of more frequently diagnosed groups.

**Supplementary Information:**

The online version contains supplementary material available at https://doi.org/10.1186/s13034-026-01176-4.

## Background

Attention-deficit/hyperactivity disorder (ADHD) is one of the most commonly diagnosed psychiatric conditions in children and adolescents [1]. Its prevalence varies markedly across socio-demographic groups: boys are diagnosed more often than girls [2], children with lower socio-economic status (SES) more often than their higher SES peers [3], and native-born children typically more often than those with migration backgrounds [4, 5]. While these patterns are well documented, their causes are not fully understood. In the absence of biomarkers, ADHD diagnosis relies on the assessment of behavioural symptoms, as reported by parents, teachers, and other informants. These symptoms exist on a continuum with no clear-cut boundary separating clinical from non-clinical cases [6, 7]. Thus, diagnosis inevitably involves a degree of interpretation, leaving room for contextual factors to influence clinical outcomes [8, 9]. For clarity, ADHD will therefore be used to denote the neurodevelopmental condition as such, and ADHD diagnosis to denote the formal clinical recognition of that condition. The focus of, and the outcome in, the present paper is on the diagnosis.

Gender is the most well-researched socio-demographic dimension in relation to ADHD, with boys consistently exhibiting a higher prevalence [10, 11]. However, the male-to-female ratio tends to be higher in clinical than in representative population samples, suggesting that the observed gap may partly reflect under-identification of girls [2, 12]. Socio-economic differences in ADHD are also well-researched, with a systematic review concluding that 35 of 42 studies reported higher diagnostic rates or levels of symptoms in lower SES groups [3]. As for differences by migration background, studies from most high-income countries show that diagnostic rates are especially low among first-generation immigrant children, but also second-generation immigrants have lower rates than native children [4, 5, 13–16]. Results concerning symptom levels are more mixed but, overall, do not suggest systematically lower rates among immigrant children [17–21]. This discrepancy between diagnostic rates and symptom levels may suggest possible under-identification of immigrant children.

One factor that has received limited attention in this context, but that may be central to understanding the socio-demographic patterns, is academic achievement. Several of the core diagnostic criteria for ADHD listed in the Diagnostic and Statistical Manual of Mental Disorders (DSM-5), especially those related to inattention, explicitly refer to school functioning (e.g., making careless mistakes in schoolwork, failing to finish assignments, having difficulty sustaining attention during lessons). School is also identified in the DSM-5 as one of the key settings in which symptoms should be present and cause impairment [22].

From this perspective, academic achievement may be seen as a partial proxy for the severity of underlying symptoms, especially those related to inattention: ADHD directly impair learning and academic achievement, and ADHD is among the psychiatric or neurodevelopmental conditions that are most strongly associated with low achievement [23, 24]. Because ADHD impair academic functioning, academic underachievement may act as a signal that triggers clinical referral and diagnostic investigation. Research also shows that reduced academic functioning is among the most common reasons that children come to clinical attention and that teachers and other school staff are often the first to suggest an ADHD diagnosis in children [25–28]. Moreover, Swedish healthcare providers report that ADHD is the single most common reason for school referrals to specialised child and adolescent psychiatry [29]. Since academic achievement also varies along the same socio-demographic dimensions as ADHD—gender, SES, and migration background—this suggests that socio-demographic differences in ADHD diagnosis may partly reflect underlying differences in academic functioning or in ADHD-related symptoms more broadly.

For gender and SES, the direction of the achievement gradients runs parallel to the ADHD gradients: girls outperform boys academically [30] and have lower rates of diagnosed ADHD [2], while higher-SES children outperform lower-SES children [31] and are likewise diagnosed less often [3]. Thus, academic achievement could partly account for the observed gender- and SES-differences in ADHD diagnosis. For migration background, the patterns are less consistent: immigrant children typically perform worse in school, at least in European countries [32–34], but simultaneously tend to have lower rates of diagnosed ADHD than their native peers [4]. Thus, the direction of the achievement gradient runs counter to the diagnostic gradient, meaning that accounting for achievement would be expected to widen, rather than narrow, the difference in diagnosis rates. Existing research on socio-demographic differences in ADHD has rarely accounted for these sources of overlap, and it remains unknown whether or how socio-demographic differences in ADHD diagnosis are shaped by differences in academic achievement.

### The Swedish context

Sweden has a universal, tax-funded health-care system, with care provided free of charge for children. According to national guidelines [35], ADHD among children should be treated within specialised child and adolescent psychiatry. A neuropsychiatric investigation for suspected ADHD should be conducted by a multidisciplinary team including, at a minimum, a psychologist and a psychiatric specialist, and should incorporate a comprehensive functional assessment covering school, home, and social settings. Schools and school health services play a central role through early detection of suspected neurodevelopmental difficulties, and investigations should be conducted in collaboration with school staff to assess the child’s school functioning. As stated, ADHD is also the most common reason for school referrals to specialist healthcare in Sweden [29]. The guidelines further note that certain groups are at higher risk of being overlooked, including children with a first language other than Swedish, girls, and socioeconomically vulnerable children. It should, moreover, be noted that there has been a large growth in private diagnostic services in Sweden, possibly due to long waiting lists in public care [36]. Since these private services are not covered by the public health insurance, access for low-income families is restricted.

The rate of diagnosed ADHD has increased substantially among Swedish children and adolescents, reaching around 8% in 2022 [37], and Sweden now has among the highest rates of ADHD medication use per capita in Europe [38–40]. In line with international patterns, diagnostic rates are highest among boys [41] as well as lower SES and native-born children [42].

Swedish compulsory school lasts for 10 years, from grade 0 (age 6) to grade 9 (age 16). Upper secondary school is voluntary but has near 100% enrolment. Access to upper secondary schools and programmes is almost completely determined by compulsory school-leaving grades, making these grades very high stakes for pupils.

### The present study

Focussing on Swedish compulsory school pupils, the aim of this study was to investigate how socio-demographic (gender, SES, migration background) differences in ADHD diagnosis change when differences in academic achievement are taken into account.

Since low academic achievement is a key marker of ADHD-related impairment in school settings, comparisons of socio-demographic gradients in diagnosis at given levels of achievement may indicate the degree to which access to diagnosis and care differs between groups. Sweden, with its universal health-care system and national registers covering both diagnostic and educational outcomes for the full population, provides a substantively and methodologically important setting for examining these questions.

## Methods

### Data and population

This is a population-based cross-sectional study using linked national registers in Sweden. Individual-level data from several administrative registers were linked using the personal identification numbers assigned to all Swedish residents: the National Patient Register [43] and the Prescribed Drugs Register [44], maintained by the National Board of Health and Welfare; school and pupil registers maintained by the Swedish National Agency for Education [45]; and the Total Population Register [46] and the Longitudinal Integrated Database for Health Insurance and Labour Market Studies (LISA) [47], maintained by Statistics Sweden.

The study population comprises all pupils who graduated from the final year of compulsory school (grade 9) between 2015 and 2017. Pupils with missing data on parental education (*n* = 4212) or migration background (country of birth; *n* = 4) were excluded. The final analytical sample comprised 296,380 pupils (mean age = 16 years).

### Measures

*ADHD*: ADHD diagnosis was defined using International Classification of Diseases, 10th Revision (ICD-10) code F90, based on diagnoses from specialised inpatient or outpatient care recorded in the National Patient Register. Since the register has possible undercoverage of private providers [43], data from the Prescribed Drugs Register, specifically dispensations of ADHD medications (Anatomical Therapeutic Chemical (ATC) code N06B), were used as a complement. Guanfacine, used by around 10% of Swedish children receiving drug treatment for ADHD [40], is not covered by this ATC code and is therefore not included. The Prescribed Drugs Register covers all prescription drugs dispensed at Swedish pharmacies, with very low missingness [48]. About 80% of adolescents diagnosed with ADHD initiate drug treatment [41]. Pupils were coded as having an ADHD diagnosis if they received a diagnosis (ICD-10: F90) or were dispensed ADHD medication (ATC: N06B) at least once in the year before or the same year as they graduated from compulsory school. A narrow timeframe was chosen to ensure temporal correspondence between the diagnostic and achievement measures, and to capture pupils whose ADHD was likely to be affecting their current school functioning.

#### Socio-demographic characteristics

Gender was measured as the pupil’s legal sex (girl/boy). SES was measured as the highest attained education of either parent (compulsory school or less, upper secondary school, tertiary education < 3 years, tertiary education ≥ 3 years). Migration background was classified as native (pupil and at least one parent born in Sweden), second-generation immigrant (pupil born in Sweden, both parents foreign-born), and first-generation immigrant (pupil foreign-born).

#### Academic achievement

Academic achievement was measured by compulsory school-leaving grades. At graduation, pupils are graded in 16 subjects, with grades ranging from F (“fail”; 0 credits) to A (20 credits). The subject grades are summed to produce an overall grade total (range 0–320). Grades are intended to reflect the degree to which pupils’ performance corresponds to national learning standards; however, teachers have considerable discretion, and research indicates that teachers also consider pupils’ motivation and behaviour when assigning grades [49]. Grades may therefore capture aspects of school functioning beyond learning, including behavioural difficulties associated with ADHD. In this study, both the raw grade total and the within-graduation-year percentile rank were used; the latter to account for grade inflation over time.

*Covariates*: The aim of the analysis was descriptive: to characterise how socio-demographic differences in ADHD diagnosis change when taking academic achievement into account. The study did not aim to estimate causal effects of achievement on diagnosis. Covariates were nonetheless included to account for known sources of variation that would otherwise complicate interpretation of the observed patterns. Adjustment for graduation year accounted for the marked increase in ADHD diagnostic rates over the study period [37, 40], ensuring that associations were not driven by temporal trends in prevalence. Pupil age was included because immigrant pupils are more often older than the modal age given the educational stage, and age itself is associated with ADHD diagnosis [41]. Birth month was included to account for well-documented relative age effects, whereby children born later in the academic year are more likely to be diagnosed with ADHD [50].

### Statistical analysis

The following logistic regression model was fitted:

$$\begin{array}{l}\:\mathrm{log}\left(\frac{P\left(ADH{D}_{i}=1\right)}{1-\:P\left(ADH{D}_{i}=1\right)}\right)={{\upbeta}}_{0}+{{\upbeta\:}}_{1}{\mathrm{G}\mathrm{e}\mathrm{n}\mathrm{d}\mathrm{e}\mathrm{r}}_{\mathrm{i}}\\ \quad+{{\upbeta}}_{2}{\mathrm{P}\mathrm{a}\mathrm{r}\mathrm{E}\mathrm{d}\mathrm{u}}_{\mathrm{i}} +{{\upbeta}}_{3}{\mathrm{M}\mathrm{i}\mathrm{g}}_{\mathrm{i}}+{{\upbeta}}_{4}{\mathrm{A}\mathrm{c}\mathrm{h}}_{\mathrm{i}}+{{{\rm\:X}}}_{\mathrm{i}}^{\prime}{\upgamma}\end{array}$$

Where *i* denotes pupils, $\:{\upbeta\:}$ denotes coefficients, *Gender* represents gender, *ParEdu* represents a set of indicator variables for parental education, *Mig* represents a set of indicator variables for migration background, *Ach* represents academic achievement (a vector comprising the raw grade total and the within-graduation-year percentile rank), *X* represents a vector of covariates (graduation year, age, and birth month), and γ the corresponding vector of coefficients. The variables were entered in a stepwise manner. In models 1a-c, the respective socio-demographic variables were entered separately, adjusting for the basic covariates. In model 2, the socio-demographic variables were entered together, again adjusting for the same basic covariates. Model 3 added academic achievement. The aim was addressed by comparing estimates from models 2 (not adjusted for achievement) and 3 (adjusted for achievement). The odds ratios from the logistic models were also transformed into marginal effects and predicted probabilities to allow investigation of differences on the risk difference scale and to account for the non-collapsibility of odds ratios across models with different sets of covariates [51, 52]. Additional file 1 shows a visual representation of the proposed relationships between the included variables, and how adjustment for achievement influences these relationships.

Several supplementary analyses were conducted. (i) To account for possible socio-demographic differences in medication use, ADHD diagnosis was defined solely using data from the National Patient Register (ICD-10: F90). (ii) To capture pupils with a previous diagnosis but without ongoing treatment or healthcare contact, ADHD diagnoses were measured over a period of up to five years before graduation. (iii) Separate models were estimated that adjusted for concurrent internalising (depression and anxiety) and externalising (conduct disorder) diagnoses; conditions that are commonly comorbid with ADHD and are themselves associated with academic achievement. (iv) Models stratified by migration background were estimated to account for differences in socio-economic composition across groups. (v) Moderation was investigated by stratifying models by socio-demographic characteristics and achievement level, and by including interaction terms between achievement and the socio-demographic characteristics. (vi) Robustness to missing parental education data was investigated by including missing data as a separate category. (vii) To assess robustness to socio-demographic differences in the timing of first ADHD diagnosis, models were estimated adjusting for earlier diagnostic history, and restricting the outcome to pupils with a first recorded diagnosis or medication in the last 2 years.

All analyses were conducted using Stata version 14 [53]. Statistical significance was assessed at the 5% level. Data were made available by the Umeå SIMSAM Lab [54].

## Results

Table 1 shows summary statistics for the sample. The overall prevalence of diagnosed ADHD was 5.62%. Of the sample, 51% were boys, more than half had a parent with at least some tertiary education (18% < 3 years; 37% ≥ 3 years), and almost a quarter had a migration background (10% first-generation and 13% second-generation). ADHD diagnosis was more common among boys, pupils with upper-secondary-educated parents, and native-born pupils. Academic achievement was higher among girls, pupils with more highly educated parents, and native pupils; the greatest achievement differences were related to parental education (Additional file 2).

**Table 1:** Summary statistics

	*N* (%) Mean (SD)	% ADHD diagnosis
*ADHD diagnosis*		
Yes	16 649 (5.62)	
No	279 731 (94.38)	
*Gender*		
Boy	152 477 (51.45)	7.06%
Girl	143 903 (48.55)	4.09%
*Parental education*		
Compulsory school or less	18 074 (6.10)	4.79%
Upper secondary school	115 521 (38.98)	7.50%
Tertiary < 3 years	54 274 (18.31)	4.98%
Tertiary ≥ 3 years	108 511 (36.61)	4.07%
*Migration background*		
Native	229 261 (77.35)	6.38%
2nd generation	28 921 (9.76)	3.42%
1st generation	38 198 (12.89)	2.69%
Raw grade total (range 0–320)	218.2 (67.5)	-
*Year*		
2015	95 579 (32.25)	5.07%
2016	99 221 (33.48)	5.75%
2017	101 580 (34.27)	6.01%
Age (range 13–20)	16.04 (0.28)	-
Birth month (range 1–12)	6.31 (3.38)	-

Study population includes all students graduating from compulsory school year 9 in Sweden 2015–2017. Pupils with missing data on parental education or migration background (*N* = 4,216) excluded. ADHD defined as diagnosis (ICD-10: F90) from specialised inpatient or outpatient care, or dispensation of ADHD medication (ATC: N06B), at least once in the year before or the same year as compulsory school graduation. ADHD = attention-deficit/hyperactivity disorder; SD = standard deviation

**Table 2:** Logistic regression models with attention-deficit/hyperactivity disorder diagnosis as the outcome

	Model 1a OR[95% CI]	Model 1b OR[95% CI]	Model 1c OR[95% CI]	Model 2 OR[95% CI]	Model 3 OR[95% CI]
*Gender (ref: girl)*					
Boy	1.74***			1.73***	1.21***
	[1.69,1.80]			[1.67,1.79]	[1.17,1.25]
*Parental education (ref: compulsory)*					
Upper secondary		2.06***		1.15***	1.60***
		[1.91,2.22]		[1.06,1.24]	[1.48,1.74]
Tertiary < 3 years		1.36***		0.77***	1.72***
		[1.26,1.48]		[0.70,0.83]	[1.58,1.87]
Tertiary ≥ 3 years		1.12**		0.62***	2.00***
		[1.04,1.21]		[0.57,0.67]	[1.84,2.18]
*Migration background (ref: native)*					
2nd generation			0.50***	0.47***	0.43***
			[0.47,0.53]	[0.44,0.50]	[0.40,0.45]
1st generation			0.24***	0.25***	0.18***
			[0.23,0.26]	[0.23,0.27]	[0.16,0.19]
Grade percentile					0.96***
					[0.96,0.96]
Raw grade total					0.99***
					[0.99,0.99]
Pseudo R^2^	0.02	0.02	0.03	0.05	0.17
N	296,380	296,380	296,380	296,380	296,380

*p < 0.05, **p < 0.01, ***p < 0.001. DHD = attention-deficit/hyperactivity disorder; OR = odds ratio; CI = confidence interval. Models 1a-1c adjusted for year, age, and birth month. Model 2: adjusted for year, age, and birth month + gender, parental education and migration background (simultaneously). Model 3: adjusted for year, age, and birth month + gender, parental education and migration background + raw grade total and percentile rank

Table 2 presents the main estimates, beginning with results when each socio-demographic characteristic was entered separately (models 1a-c). Boys had higher odds of ADHD diagnosis than girls (OR = 1.74; 95%CI[1.69,1.80]). There was a non-linear relationship between parental education and ADHD diagnosis: the lowest odds were among pupils whose parents had at most compulsory school education (reference category), followed by pupils with tertiary-educated parents (< 3 years: OR = 1.36; 95%CI[1.26,1.48]; ≥ 3 years: OR = 1.12; 95%CI[1.04,1.21]), with the highest odds observed among pupils whose parents had at most upper-secondary education (OR = 2.06; 95%CI[1.91,2.22]). Second-generation, and especially first-generation, immigrants had lower odds than native pupils (second generation: OR = 0.50; 95%CI[0.47,0.53]; first generation: OR = 0.24; 95%CI[0.23,0.26]). The patterns for gender and migration background remained stable when all socio-demographic variables were entered simultaneously (model 2). However, the SES gradient partially reversed, with the lowest odds now observed among pupils with tertiary-educated parents (< 3 years: OR = 0.77; 95%CI[0.70,0.83]; ≥ 3 years: OR = 0.62; 95%CI[0.57,0.67]). This reversal reflects the fact that parents of immigrant pupils have lower education on average and are particularly overrepresented in the compulsory school or less-category (see Additional file 2).

Adjustment for achievement (model 3) affected all three socio-demographic gradients. The excess odds among boys were strongly reduced, from OR = 1.73 to OR = 1.21 (95%CI[1.17,1.25]). The SES-gradient reversed again, with the highest odds now observed among children of tertiary-educated parents (< 3 years: OR = 1.72; 95%CI[1.58,1.87]; ≥ 3 years: OR = 2.00; 95%CI[1.84,2.18]). The already lower odds among immigrant pupils decreased further (second generation: OR = 0.43; 95%CI[0.40,0.45]; first generation: OR = 0.18; 95%CI[0.16,0.19]). Post-hoc Wald tests confirmed that the differences between models 2 and 3 were statistically significant for all socio-demographic coefficients.

**Table 3:** Marginal effects of socio-demographic characteristics on attention-deficit/hyperactivity disorder diagnosis

	Model 2 Marginal effect [95% CI]	Model 3 Marginal effect [95% CI]
*Gender (ref: girl)*		
Boy	0.028***	0.009***
	[0.026,0.029]	[0.007,0.011]
*Parental education (ref: compulsory)*		
Upper secondary	0.009***	0.018***
	[0.004,0.013]	[0.016,0.021]
Tertiary < 3 years	-0.014***	0.022***
	[-0.019,-0.009]	[0.019,0.025]
Tertiary ≥ 3 years	-0.023***	0.030***
	[-0.028,-0.019]	[0.027,0.033]
*Migration background (ref: native)*		
2nd generation	-0.035***	-0.037***
	[-0.037,-0.032]	[-0.040,-0.035]
1st generation	-0.050***	-0.057***
	[-0.052,-0.048]	[-0.059,-0.055]
N	296,380	296,380

* *p* < 0.05, ** *p* < 0.01, *** *p* < 0.001. CI = confidence interval. Model 2: adjusted for year, age, and birth month + gender, parental education and migration background. Model 3: adjusted for year, age, and birth month + gender, parental education and migration background + raw grade total and percentile rank

To facilitate interpretation of the magnitude of these changes, risk differences (marginal effects) are presented in Table 3. The excess risk among boys was reduced by approximately two thirds, from 2.8% points to 0.9% points. The difference comparing children of tertiary and compulsory educated parents changed from negative to positive: from − 1.4 to + 2.2% points (< 3 years) and from − 2.3 to + 3.0% points (≥ 3 years), respectively. The lower diagnostic probability among immigrant pupils decreased further, though only slightly: from − 3.5 to − 3.7% points (second generation) and from − 5.0 to − 5.7% points (first generation), respectively.

**Fig. 1:**
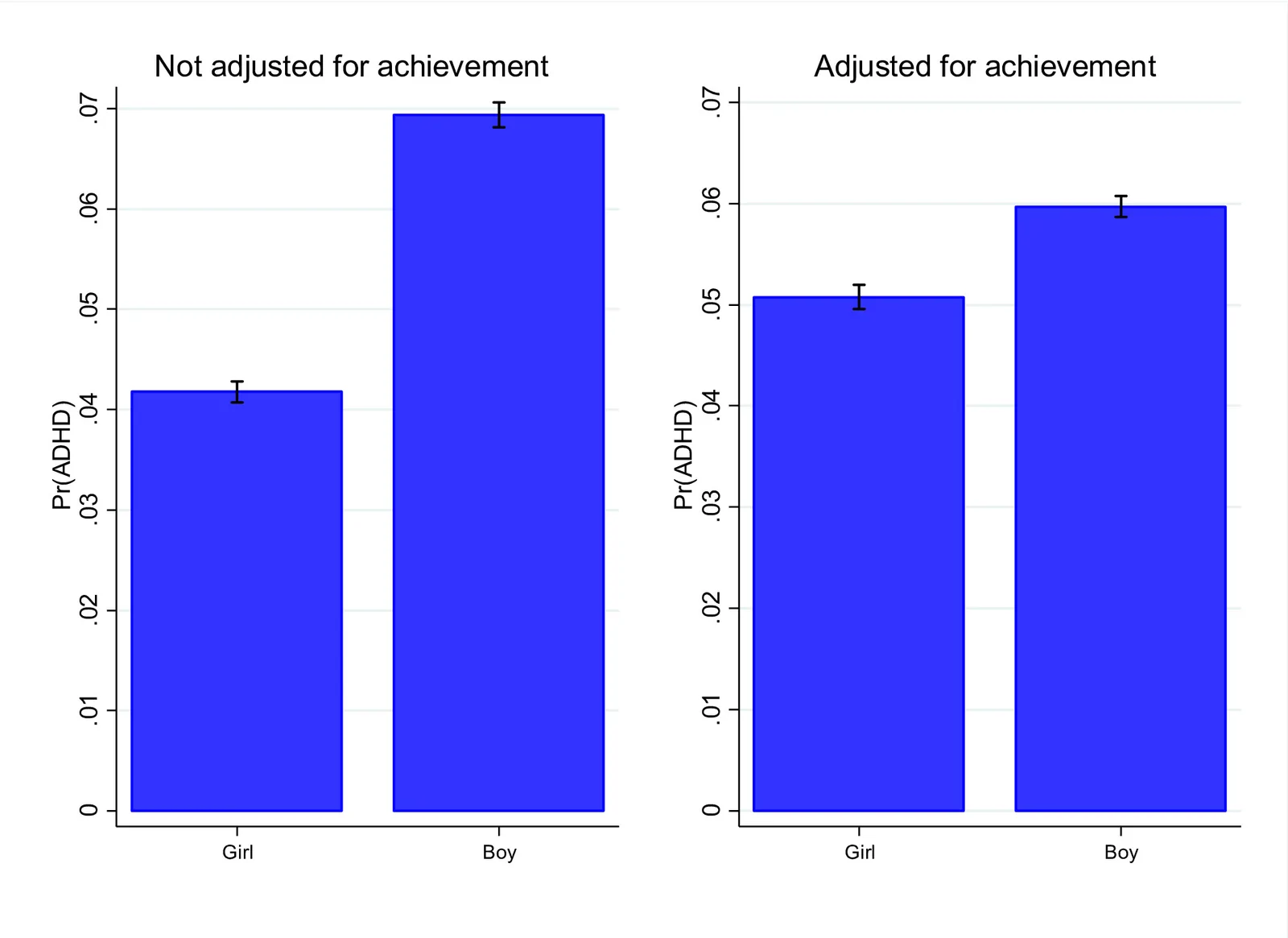
Predicted probability of attention-deficit/hyperactivity disorder diagnosis by gender. Predicted probabilities of ADHD diagnosis from logistic regression models. Left panel: adjusted for year, age, birth month, parental education and migration background (model 2). Right panel: adjusted for year, age, birth month, parental education, migration background, and raw grade total and percentile rank (model 3). Error bars indicate 95% confidence intervals. *N* = 296,380

**Fig. 2:**
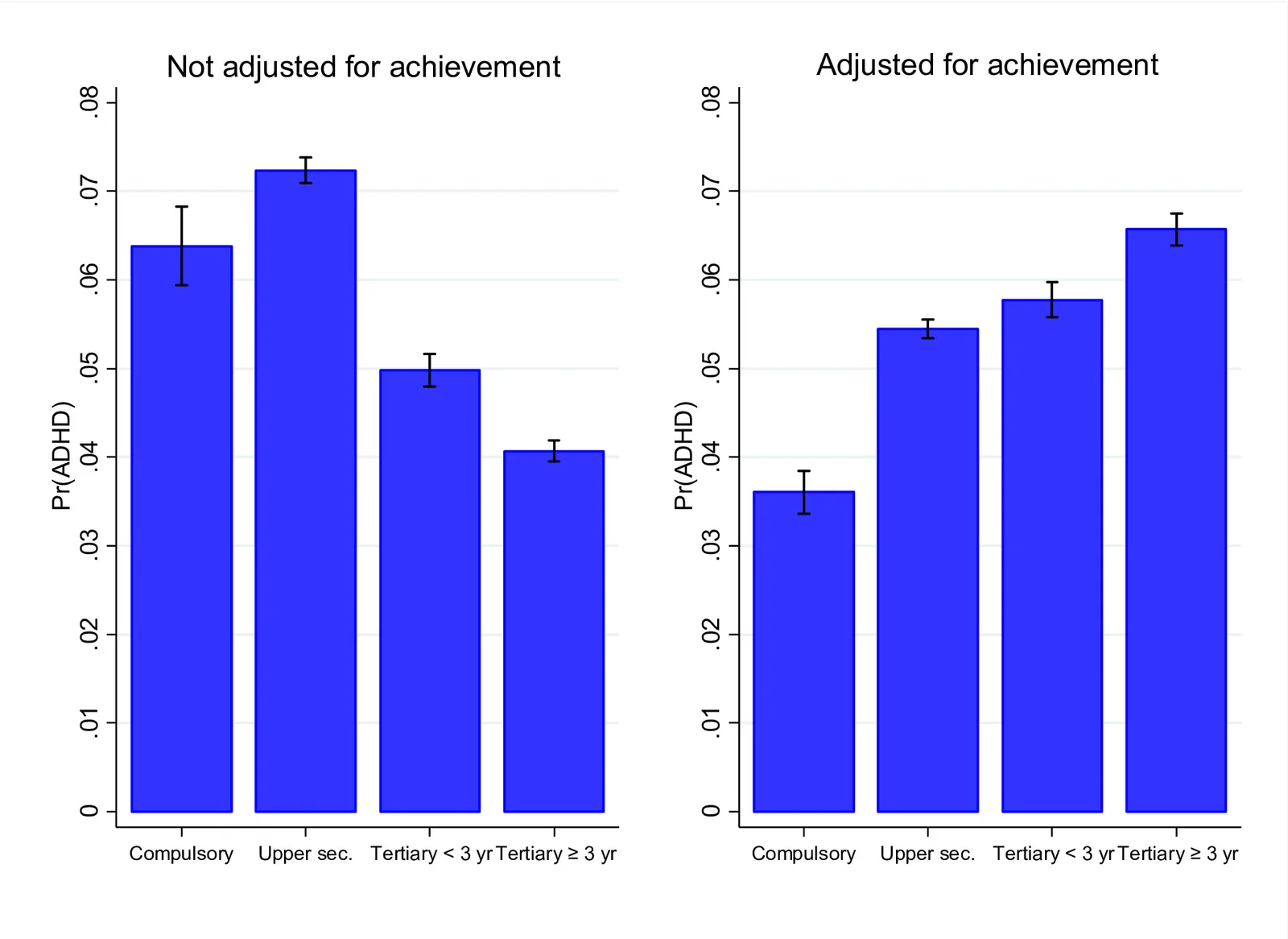
Predicted probability of attention-deficit/hyperactivity disorder diagnosis by parental education. Predicted probabilities of ADHD diagnosis from logistic regression models. Left panel: adjusted for year, age, birth month, gender and migration background (model 2). Right panel: adjusted for year, age, birth month, gender, migration background, and raw grade total and percentile rank (model 3). Error bars indicate 95% confidence intervals. *N* = 296,380

**Fig. 3:**
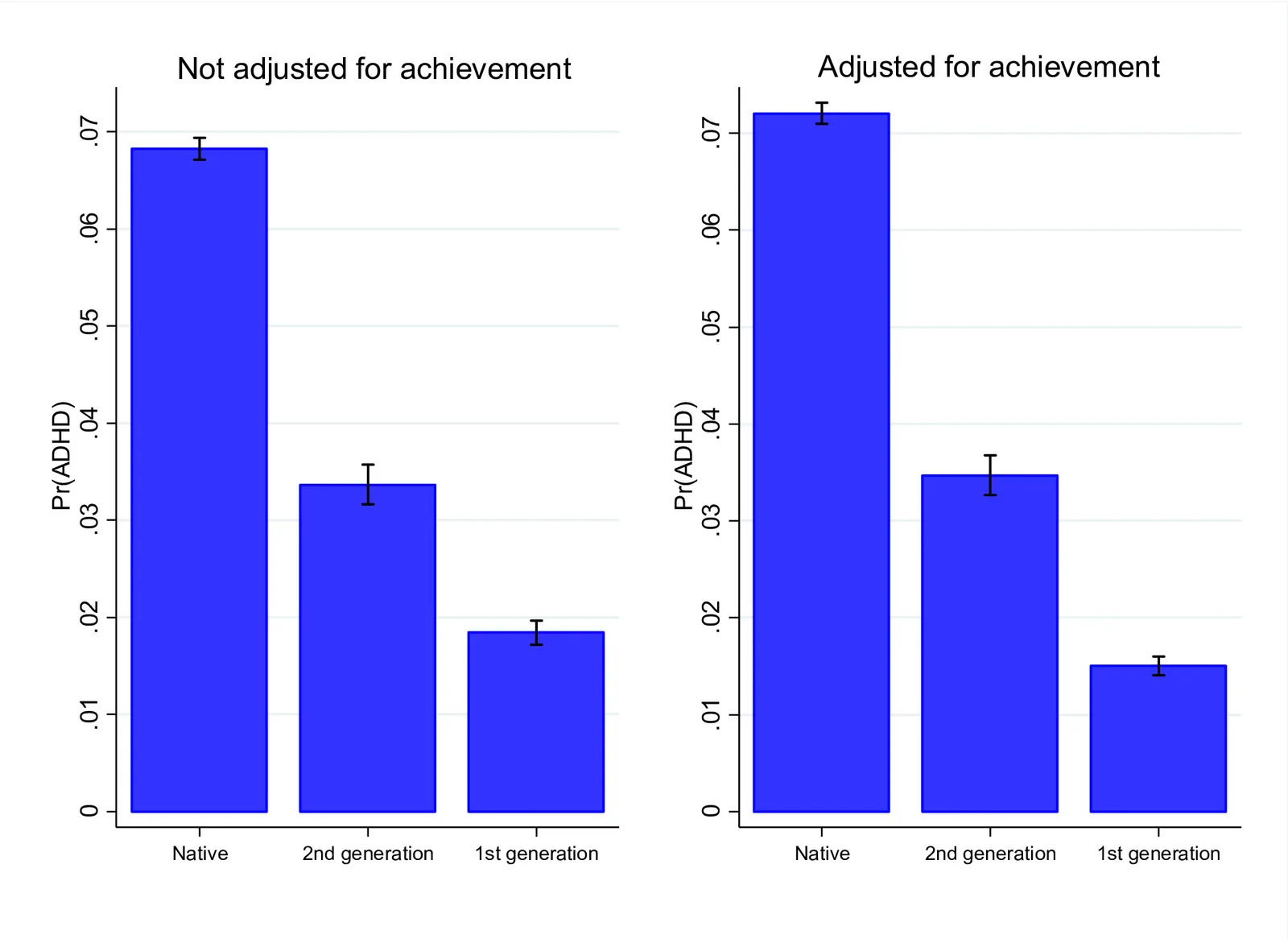
Predicted probability of attention-deficit/hyperactivity disorder diagnosis by migration background. Predicted probabilities of ADHD diagnosis from logistic regression models. Left panel: adjusted for year, age, birth month, gender and parental education (model 2). Right panel: adjusted for year, age, birth month, gender, parental education, and raw grade total and percentile rank (model 3). Error bars indicate 95% confidence intervals. *N* = 296,380

Figures 1, 2 and 3 illustrate the results by showing predicted probabilities of ADHD diagnosis (full tables in Additional file 3). The gender gap narrowed considerably, the SES-gradient partly reversed, and the differences between native and second-generation immigrant pupils became somewhat larger, after adjustment for achievement.

Supplementary analyses showed that the results were substantively similar when using only data from the National Patient Register to define ADHD diagnosis (Additional file 4), when extending the diagnostic window to up to five years prior to graduation (Additional file 5), and when adjusting for concurrent internalising (depression and anxiety disorders) and externalising (conduct disorder) diagnoses (Additional file 6). However, the reduction in the gender gap was less marked in the comorbidity-adjusted analyses. Results stratified by migration background showed that the reduction of the gender gap after adjustment for academic achievement was most pronounced among native pupils. The SES reversal was also most pronounced among native pupils, where the gradient shifted from strongly negative to positive. Among first-generation immigrant pupils, higher parental education was associated with higher odds of ADHD diagnosis even before adjustment, and this pattern was further amplified after accounting for achievement (Additional file 7). Moderation analyses showed that the association between achievement and ADHD diagnosis was stronger among native than among immigrant pupils (Additional file 8). Results were robust to the inclusion of missing parental education as a separate category (Additional file 9). When accounting for diagnostic timing by adjusting for earlier diagnostic history or restricting the outcome to pupils with a recent first diagnosis, results for SES and migration background were similar to the main results. Accounting for diagnostic timing substantially attenuated the gender gap before adjustment for achievement, from OR = 1.73 to OR = 1.23 (adjusting for diagnostic history) and OR = 1.22 (restricting the outcome to recent first diagnoses) (Additional file 10). This is consistent with the well-documented later average diagnosis of girls [37]. After adjustment for achievement, the gender gap even reversed, to OR = 0.89 and OR = 0.84 respectively, meaning that that girls had higher odds of diagnosis than boys once both achievement and diagnostic timing was accounted for.

## Discussion

Focussing on Swedish compulsory school pupils, the aim of this study was to investigate how socio-demographic (gender, SES, migration background) differences in ADHD diagnosis change when differences in academic achievement are taken into account. Results showed that adjustment for achievement reduced the gender gap by approximately two thirds and reversed the SES-gradient: from higher odds among pupils with less highly educated parents (compulsory or upper-secondary education) to higher odds among pupils with tertiary-educated parents. In contrast, the already substantial differences between native and immigrant, especially first-generation immigrant, pupils persisted, and even widened slightly, after adjustment for academic achievement. The overarching finding is thus that the socio-demographic patterning of ADHD diagnosis is conditional on academic achievement: when comparing pupils with similar levels of achievement, the familiar socio-demographic patterns change, sometimes substantially.

These patterns can be understood in light of the two-stage process outlined in the background: ADHD impair academic achievement [24], and underachievement in turn can act as a signal that triggers referral and diagnosis [26, 27]. Moreover, school-related difficulties figure prominently in the diagnostic criteria for ADHD, especially those related to inattention, and school is emphasised as a key setting in which symptoms should be present and cause impairment. To the extent that achievement reflects underlying symptom severity, socio-demographic differences in diagnosis net of achievement may indicate that some groups are less likely to receive a diagnosis even at comparable levels of (academic) impairment.

For gender and SES, the changes in the diagnostic gradients after adjustment for achievement were in the expected direction: girls and high-SES children perform better in school [30, 31], and adjustment for achievement, which is negatively associated with ADHD diagnosis, therefore narrowed (gender) or even reversed (SES) the differences in diagnostic probability. Put differently, the higher diagnostic rates among boys and low-SES children may be partly attributable to academic underachievement, and to a higher prevalence of ADHD symptoms contributing to underachievement, in these groups.

Nonetheless, while adjustment for achievement accounted for approximately two thirds of the gender gap—in line with evidence that boys experience more, and more severe, ADHD symptoms [2]—the remaining third could not be attributed to differences in achievement. This remaining gap may reflect residual gender differences in ADHD symptoms that are unrelated to academic achievement. It may also reflect differential referral or diagnosis. Girls disproportionately exhibit inattentive symptoms [12], and research indicates that teachers are less likely to identify pupils as having ADHD or recommend referral when pupils exhibit inattentive rather than hyperactive or impulsive symptoms [55, 56]. Inattentive symptoms may be less visible but are simultaneously more strongly associated with low achievement [57, 58]. Thus, teachers or other school staff may be less likely to interpret underachievement among girls as a sign of ADHD, and consequently less likely to recommend referral. It has also been suggested that girls more successfully compensate for or mask ADHD-related difficulties through prosocial behaviours [59–61], which may further reduce the likelihood of detection and referral. There is also some evidence of differential diagnosis by gender: a vignette study showed that clinicians were more likely to suggest ADHD in boys exhibiting subthreshold symptoms [62], while a register-survey study found that the association between symptom severity and diagnosis was stronger among girls, possibly indicating a higher diagnostic threshold [63]. However, these suggested explanations should be seen in the context of the supplementary analyses accounting for diagnostic timing (Additional file 10). When adjusting for diagnostic history or restricting the outcome to recent first diagnoses, the achievement-adjusted gender gap not only narrowed but reversed, with girls having higher odds than boys. These supplementary findings suggest that much of the remaining (achievement-adjusted) excess rates among boys observed in the main analysis reflects the fact that boys are diagnosed at a younger age than girls [37]. Thus, rather than a persistent under-identification of girls, due to continued differential referral or diagnosis, this suggests that, in adolescence, previously undiagnosed girls have a higher probability of diagnosis at a given level of academic achievement. One hypothesis is that academic underachievement is less normative among girls, and thus more likely to be interpreted as reflecting an underlying disability rather than, for instance, disengagement and low motivation.

As for SES, lower SES children generally exhibit more ADHD symptoms [3, 64], which may explain the negative gradient in models not adjusting for achievement. Since lower-SES children also perform worse in school, it was expected that the SES-gradient would narrow when achievement was held constant. However, a reversal of the gradient, with the highest risks observed in high-SES children, was not anticipated. Compared with gender, there is less research on socio-economic differences in referrals or diagnosis. One British study found no SES-gradient in diagnosis after adjustment for differences in reported symptoms, suggesting an absence of clinical bias [64]. A few studies have compared parent- and teacher-reported ADHD symptoms across SES groups, but with mixed results: one study found higher levels of ADHD symptoms among low-SES children when reported by teachers but not by parents [65], while another found the opposite [66]. Sociological research on SES and schooling may, however, help inform the interpretation of the reversed SES gradient. First, higher-SES parents have greater health literacy [67] and may be more likely to recognise children’s difficulties as symptoms of ADHD. Second, higher-SES parents have higher educational aspirations and expectations [68], and are more likely to respond to academic underachievement among their children through compensatory strategies [69]. Such strategies may include seeking diagnosis and treatment, or pursuing special educational support, which in turn may be easier if the child has a neuropsychiatric diagnosis [70]. Third, once the difficulties and the need for compensatory strategies have been identified, higher-SES parents may more successfully navigate the school and healthcare systems to obtain support for their children [71]. Qualitative studies show that the diagnostic process can be prolonged and arduous, involving interactions with multiple professionals and organisations [72, 73] as well as long waiting lists in public healthcare [36]. Greater resources in terms of knowledge, networks, or financial means may be advantageous in navigating such processes [74].

The results concerning migration background were largely in line with expectations. Immigrant children in Sweden and most European countries generally perform worse in school [32–34], but simultaneously have lower rates of ADHD diagnosis [4]. Accordingly, and in contrast with the results for gender and SES, the lower odds among immigrant compared with native pupils persisted and even became somewhat more pronounced after adjustment for academic achievement. The persistence of these differences, also when comparing pupils at similar levels of achievement, may indicate barriers to accessing psychiatric services, such as language difficulties, lower familiarity with the school and healthcare system, or stigma [75–77]. The persistence may also reflect differential detection by parents (e.g., due to differing understandings of mental health) or teachers (e.g., due to differing expectations of children’s behaviour) [78]. Existing European research on symptom levels by migration background has yielded mixed results—with some studies finding higher [17] and others lower [20] levels of symptoms among immigrant children, and still others finding no differences [18, 21]—but does not suggest a substantially lower symptom burden among immigrant children.

More generally, besides reflecting differential referral or diagnosis, the patterns described above are also consistent with the possibility that low academic achievement has different underlying causes in different socio-demographic groups. Low achievement among high-SES and native pupils may more often reflect neurodevelopmental difficulties such as ADHD per se, whereas low achievement among low-SES or immigrant children may more often be attributable to other factors such as limited resources or language barriers. This interpretation is partly supported by supplementary analyses showing that the negative association between achievement and ADHD diagnosis was stronger among native than immigrant pupils. Such compositional differences would be expected to change the observed socio-demographic gradients when achievement is held constant, even in the absence of any differences in diagnostic detection or practice.

### Limitations

Several limitations should be noted. First, no data on ADHD symptoms are available in population registers. While academic achievement is associated with symptom severity, it is far from a perfect proxy. Children with similar levels of symptoms may differ substantially in terms of academic achievement, and low achievement can reflect many factors unrelated to ADHD. It therefore cannot be determined whether the socio-demographic patterns observed net of achievement reflect residual differences in underlying symptom levels or differences in the likelihood of referral or diagnosis at a given level of symptoms. Likewise, since parental ADHD may contribute to both lower parental education and higher rates of ADHD in children [79], the SES gradients in both ADHD diagnosis and achievement partly reflect unmeasured heritability. However, the study did not intend to estimate the “true” socio-demographic gradients in ADHD, since this would require extensive data on symptoms, but to describe socio-demographic differences in diagnosis among pupils at similar levels of academic functioning (as measured by academic achievement).

Second, grades were measured at the end of compulsory school, while many children receive their ADHD diagnosis several years earlier. Achievement and diagnosis may therefore have influenced each other in ways that cannot be disentangled with the cross-sectional design employed here. Given that the present findings concerning gender differences were sensitive to differential diagnostic timing, this limitation is particularly relevant in relation to gender. However, the study did not aim to estimate causal effects of achievement on diagnosis or vice versa. Nonetheless, longitudinal data would be needed to capture the sequential process linking symptoms, achievement, and diagnosis over time.

Third, ADHD diagnosis was defined using specialist diagnoses recorded in the National Patient Register, which only partially covers private healthcare providers and may be subject to underreporting. This could lead to underestimation of the prevalence, particularly among groups with greater access to private care. Fourth, the broad categorisation into native, second-generation and first-generation immigrants masks important within-group heterogeneity in terms of language or culture. Parental education may also be measured with greater error among immigrant parents, as foreign qualifications may not be accurately captured in Swedish registers. Fifth, the diagnostic process itself was not observed, meaning that it cannot be determined to whether the observed patterns are driven by differences in parental help-seeking, school referrals, clinical decision-making, or some combination of these.

## Conclusions

The socio-demographic patterning of ADHD diagnosis among Swedish compulsory school pupils looks substantially different when academic achievement is taken into account. The gender gap narrows considerably, the SES gradient reverses, and the differences between native and immigrant pupils persist or widen. When also accounting for differential timing of diagnosis, the gender gap may even reverse, with higher rates among girls. These findings suggest that socio-demographic differences in ADHD diagnosis are partly shaped by differences in academic achievement, but that the role of achievement differs across socio-demographic dimensions.

The results have implications for policy and practice by underscoring the need to ensure equitable access to diagnostic services and healthcare. Lower diagnostic rates among underachieving girls, lower-SES children, and, especially, immigrant children may indicate entrenched barriers related to detection, referral, or diagnosis. Ensuring that struggling children are identified and supported regardless of their social and demographic characteristics is a concern for both healthcare services, which are responsible for diagnosis and treatment, and schools, which play a central role in the detection and referral of children with suspected ADHD.

Future research should use longitudinal data to track the emergence and development of these socio-demographic differences, and the role of academic achievement in shaping them, as pupils progress through the education system. Longitudinal studies using data from schools as well as healthcare providers could also follow the full diagnostic process—from initial detection of difficulties, through referral, to clinical assessment and diagnosis—and thereby illuminate at which stage in the process that socio-demographic differences arise. For instance, given that the observed gender gap appears sensitive to differential diagnostic timing, this would clarify to what extent this gap reflects delays in diagnosis among girls, and when during the academic career that achievement contributes most to narrowing or even reversing the gap. Furthermore, studies with access to data on ADHD symptoms could investigate whether similar patterns emerge when symptom severity is held constant, which would help distinguish between differential referral or diagnostic practices on the one hand, and socio-demographic differences in the composition of low-achieving pupils on the other. Finally, cross-country comparisons could reveal whether the patterns observed here are specific to the Swedish context—with its universal public healthcare, reliance on psychiatrists or related specialists for diagnosis, and particular school structure—or whether they generalise to other institutional settings.

## Supplementary Information

Below is the link to the electronic supplementary material.

Supplementary Material 1. Additional file 1. Directed acyclic graph. Directed acyclic graph illustrating the assumed relationships between sociodemographic characteristics, ADHD, academic achievement, and ADHD diagnosis, including a description of which pathways are blocked and which remain open after adjustment for academic achievement. Additional file 2. Summary statistics on socio-demographic characteristics and academic achievement. Tables showing socio-demographic differences in academic achievement and the distribution of parental education across migration background categories. Additional file 3. Predicted probabilities of ADHD diagnosis underlying Figures 1–3. Table presenting the exact predicted probabilities of ADHD diagnosis from logistic regression models, with and without adjustment for academic achievement, by gender, parental education, and migration background. Additional file 4. Sensitivity analysis defining ADHD diagnosis using only diagnoses from the National Patient Register. Logistic regression results when ADHD diagnosis is defined solely using ICD-10 code F90 (excluding pupils identified through dispensed ADHD medication only), motivated by socio-demographic differences in medication use given diagnosis. Includes a cross-tabulation of pupils with ADHD diagnoses and pharmacological treatment. Additional file 5. Sensitivity analysis with extended diagnostic window. Logistic regression results when ADHD diagnosis is defined as any specialised inpatient or outpatient care visit with an F90 code in the five years prior to, or the same year as, compulsory school graduation. Additional file 6. Sensitivity analysis adjusting for psychiatric comorbidity. Logistic regression results with additional adjustment for depression and anxiety disorders (ICD-10: F30–F48; ATC: N05B, N05C, N06A) and conduct disorder (ICD-10: F91), conditions that are commonly comorbid with ADHD and themselves associated with academic achievement. Additional file 7. Models stratified by migration background. Logistic regression results separately estimated for native, second-generation immigrant, and first-generation immigrant pupils, examining whether socio-demographic gradients and the effect of adjustment for achievement differ across these groups. Additional file 8. Moderation analyses. Results from moderation analyses examining (i) whether the association between academic achievement and ADHD diagnosis differs by gender, parental education, or migration background, and (ii) whether socio-demographic differences in ADHD diagnosis differ across achievement deciles. Additional file 9. Missing parental education as separate category. Results from logistic regression models, estimated on the full sample with missing parental education data retained as a separate category rather than excluded. Additional file 10. Robustness to diagnostic timing. Results from analyses addressing potential sociodemographic differences in the timing of first ADHD diagnosis: (i) models adjusting for earlier ADHD diagnosis, and (ii) models in which the outcome is restricted to pupils with a first recorded diagnosis or medication dispensation in the last two years.

## Data Availability

The data that support the findings of this study are available from the Umeå SIMSAM Lab but restrictions apply to the availability of these data, which were used under license for the current study, and so are not publicly available. Data are, however, available through application to the Umeå SIMSAM Lab (https://www.umu.se/forskning/infrastruktur/umea-simsam-lab/).

## Abbreviations

ADHD: Attention-deficit/hyperactivity disorder
SES: Socioeconomic status
ICD-10: International Classification of Diseases, 10th Revision
DSM: Diagnostic and Statistical Manual of Mental Disorders
ATC: Anatomical therapeutic chemical
OR: Odds ratio
CI: Confidence interval
LISA: Longitudinal Integrated Database for Health Insurance and Labour Market Studies
