# Supplementary material for: Academic achievement and socio-demographic differences in ADHD diagnosis: a population-based study of Swedish compulsory school pupils
Source: Child Adolesc Psychiatry Ment Health. 2026 Sep 9;20:133. doi: 10.1186/s13034-026-01176-4 (PMC13613661; doi:10.1186/s13034-026-01176-4)
Supplement: Supplementary file 1 [file 13034_2026_1176_MOESM1_ESM.zip › Additional file 6.docx]

**Additional file 6. Adjustment for psychiatric comorbidity**

Additional file 6 shows results with adjustment for depression and anxiety disorders and conduct disorders. Depression and anxiety disorders are measured using diagnoses from specialised inpatient or outpatient care (ICD-10: F30-F39; F40-F48) and drug dispensations (ATC: NB05B, NB05C, and NB06A). Conduct disorders are measured using diagnoses from specialised inpatient or outpatient care (ICD-10: F91). These conditions are comorbid with ADHD and are themselves associated with academic achievement. Holding them constant therefore isolates the part of the achievement–ADHD association that is not attributable to shared psychiatric morbidity. However, it should also be noted that there is evidence that ADHD can cause depression (Riglin et al., 2021) and anxiety (Gair et al., 2021). Moreover, there is evidence that some children may receive other internalising or externalising diagnoses instead of, or before, an ADHD diagnosis (Quinn & Madhoo, 2014).

The results shows that the overall pattern of changes after adjustment for academic achievement is similar to the main results. A partial exception is the gender gap, which is reduced less – from an excess odds among boys of 2.57 to 1.84 – when comorbidity is held constant.

*References*

Gair, S. L., Brown, H. R., Kang, S., Grabell, A. S., & Harvey, E. A. (2021). Early Development of Comorbidity Between Symptoms of ADHD and Anxiety. *Research on Child and Adolescent Psychopathology*, *49*(3), 311-323. <https://doi.org/10.1007/s10802-020-00724-6>

Quinn, P. O., & Madhoo, M. (2014). A review of attention-deficit/hyperactivity disorder in women and girls: uncovering this hidden diagnosis. *Prim Care Companion CNS Disord*, *16*(3). <https://doi.org/10.4088/PCC.13r01596>

Riglin, L., Leppert, B., Dardani, C., Thapar, A. K., Rice, F., O'Donovan, M. C., Davey Smith, G., Stergiakouli, E., Tilling, K., & Thapar, A. (2021). ADHD and depression: investigating a causal explanation. *Psychological Medicine*, *51*(11), 1890-1897. <https://doi.org/10.1017/S0033291720000665>

**Table E1. Logistic regression models with attention-deficit/hyperactivity disorder diagnosis as the outcome. Adjustment for psychiatric comorbidity.**

|  | *Model 2* | *Model 3* |
| --- | --- | --- |
|  | *OR[95% CI]* | *OR[95% CI]* |
| *Gender (ref: girl)* |  |  |
| Boy | 2.57*** | 1.84*** |
|  | [2.48,2.67] | [1.77,1.92] |
| *Parental education (ref: compulsory)* |  |  |
| Upper secondary | 1.24*** | 1.50*** |
|  | [1.14,1.35] | [1.38,1.63] |
| Tertiary < 3 years | 0.86** | 1.48*** |
|  | [0.78,0.94] | [1.35,1.62] |
| Tertiary ≥ 3 years | 0.69*** | 1.61*** |
|  | [0.64,0.76] | [1.47,1.76] |
| *Migration background (ref: native)* |  |  |
| 2nd generation | 0.60*** | 0.56*** |
|  | [0.56,0.64] | [0.52,0.60] |
| 1st generation | 0.36*** | 0.29*** |
|  | [0.33,0.39] | [0.27,0.31] |
| Depression or anxiety disorders | 15.81*** | 9.92*** |
|  | [15.22,16.41] | [9.53,10.33] |
| Conduct disorders | 5.04*** | 3.90*** |
|  | [4.47,5.68] | [3.45,4.41] |
| Grade percentile |  | 0.96*** |
|  |  | [0.96,0.96] |
| Raw grade total |  | 0.99*** |
|  |  | [0.99,0.99] |
| N | 296380 | 296380 |

Note. * p<0.05, ** p<0.01, *** p<0.001. Abbreviations: ADHD = attention-deficit/hyperactivity disorder; OR = odds ratio; CI = confidence interval. Model 2: adjusted for year, age, and birth month + gender, parental education and migration background (simultaneously) + conduct and depression and anxiety disorders. Model 3: adjusted for year, age, and birth month + gender, parental education and migration background + conduct and depression and anxiety disorders + raw grade total and percentile rank.
