# Supplementary material for: Academic achievement and socio-demographic differences in ADHD diagnosis: a population-based study of Swedish compulsory school pupils
Source: Child Adolesc Psychiatry Ment Health. 2026 Sep 9;20:133. doi: 10.1186/s13034-026-01176-4 (PMC13613661; doi:10.1186/s13034-026-01176-4)
Supplement: Supplementary file 1 [file 13034_2026_1176_MOESM1_ESM.zip › Additional file 7.docx]

**Additional file 7. Models stratified by migration background.**

Additional file 7 shows results stratified by migration background category. This is motivated by two considerations. First, since many foreign-born parents have undergone schooling in other countries than Sweden, education may be measured with greater error among immigrants. The educational degrees of immigrants educated in another country may, moreover, not represent the same thing as corresponding Swedish degrees, and/or may not be recognised by Swedish employers. Second, there is an over-representation of boys among 1^st^ generation immigrants due to most unaccompanied minors being boys. Moreover, gender norms may differ between native and immigrant families.

Results show that the reduction of the gender gap is greater among native than immigrant pupils. The results concerning SES are more complex. In the models not adjusted for achievement, there is a clear gradient among native pupils, with strongly reduced odds among pupils with tertiary educated parents. The is no gradient among second-generation immigrants, and a reversed gradient among first-generation immigrant, with clearly elevated odds among first-generation immigrant pupils with tertiary educated parents. After adjustment for achievement, the gradient among native pupils disappears (upper secondary and < 3 years tertiary) or reverses (≥ 3 years tertiary). The non-gradient among second-generation immigrants becomes clearly positive, and the already positive gradient among first-generation immigrants becomes stronger. Thus, the overall positive gradient in the pooled sample is to an extent driven by immigrant pupils; however, the change in the gradient after adjustment for achievement is most pronounced among native pupils.

**Table F1. Logistic regression models with attention-deficit/hyperactivity disorder diagnosis as the outcome. Models stratified by migration background.**

|  | *Native* | *Native* | *2nd generation* | *2nd generation* | *1st generation* | *1st generation* |
| --- | --- | --- | --- | --- | --- | --- |
|  | *OR[95% CI]* | *OR[95% CI]* | *OR[95% CI]* | *OR[95% CI]* | *OR[95% CI]* | *OR[95% CI]* |
| Gender (ref: girl) |  |  |  |  |  |  |
| Boy | 1.70*** | 1.19*** | 1.78*** | 1.33*** | 1.81*** | 1.45*** |
|  | [1.64,1.77] | [1.15,1.24] | [1.56,2.04] | [1.16,1.53] | [1.59,2.06] | [1.27,1.66] |
| Parental education (ref: compulsory) |  |  |  |  |  |  |
| Upper secondary | 0.61*** | 1.02 | 1.22 | 1.69*** | 2.49*** | 2.89*** |
|  | [0.56,0.67] | [0.93,1.12] | [0.99,1.49] | [1.37,2.09] | [2.00,3.09] | [2.32,3.60] |
| Tertiary < 3 years | 0.39*** | 1.10 | 1.02 | 1.73*** | 3.02*** | 3.81*** |
|  | [0.35,0.43] | [0.99,1.22] | [0.80,1.31] | [1.34,2.23] | [2.39,3.80] | [3.01,4.81] |
| Tertiary ≥ 3 years | 0.30*** | 1.23*** | 0.95 | 2.18*** | 3.52*** | 5.95*** |
|  | [0.28,0.33] | [1.11,1.36] | [0.75,1.19] | [1.72,2.77] | [2.85,4.35] | [4.80,7.39] |
| Grade percentile |  | 0.96*** |  | 0.97*** |  | 0.96*** |
|  |  | [0.96,0.97] |  | [0.96,0.97] |  | [0.95,0.96] |
| Raw grade total |  | 0.99*** |  | 0.99*** |  | 1.01*** |
|  |  | [0.99,0.99] |  | [0.99,0.99] |  | [1.00,1.01] |
| Model | 2 | 3 | 2 | 3 | 2 | 3 |
| N | 229261 | 229261 | 28921 | 28921 | 38198 | 38198 |

Note. * p<0.05, ** p<0.01, *** p<0.001. Abbreviations: ADHD = attention-deficit/hyperactivity disorder; OR = odds ratio; CI = confidence interval. Model 2: adjusted for year, age, and birth month + gender, parental education and migration background (simultaneously). Model 3: adjusted for year, age, and birth month + gender, parental education and migration background + raw grade total and percentile rank.
