# Supplementary material for: Academic achievement and socio-demographic differences in ADHD diagnosis: a population-based study of Swedish compulsory school pupils
Source: Child Adolesc Psychiatry Ment Health. 2026 Sep 9;20:133. doi: 10.1186/s13034-026-01176-4 (PMC13613661; doi:10.1186/s13034-026-01176-4)
Supplement: Supplementary file 1 [file 13034_2026_1176_MOESM1_ESM.zip › Additional file 8.docx]

**Additional file 8. Moderation analyses**

Additional file 8 shows results from two types of moderation analyses: (i) associations between achievement and ADHD stratified by socio-demographic characteristics, and (ii) associations between socio-demographic characteristics stratified by achievement level (specifically grade decile).

In the first moderation analyses (Tables G1-G3), separate models are estimated for the two measures of academic achievement (the raw raw grade total and the percentile rank). Results show that neither measure show a differential association with ADHD between girls and boys, indicating that gender does not moderate the association between achievement and ADHD. There are weak and inconsistent evidence of moderation with regard to SES, in the sense that the differences between parental education groups are not linear and dependent on the measure of achievement. There is strong evidence of moderation with respect to migration background: the association between achievement and ADHD is consistently stronger among native than immigrant, especially first-generation immigrant, pupils. Interaction analyses (not shown) show that the socio-demographic differences the association between achievement and ADHD are significant for all migration background categories, for the differences between compulsory education and all other categories in models for the raw raw grade total, and for the differences between compulsory and upper secondary education in models for the percentile rank.

In the second moderation analysis (Table G4), the excess risk among boys is generally larger in the upper half of the achievement distribution, but also pronounced in the lowest decile. The SES-gradient is also generally most pronounced in the higher achievement deciles, but the low number of pupils with ADHD in these decile means that the estimates are imprecise and generally non-significant. Differences by migration background, on the other hand, are generally largest in the lowest deciles.

**Table G1. Associations between academic achievement and ADHD by gender**

|  | Girls | Boys |
| --- | --- | --- |
|  | *OR[95% CI]* | *OR[95% CI]* |
| Raw grade total | 0.987*** | 0.986*** |
|  | [0.987,0.987] | [0.986,0.987] |
| Grade percentile | 0.953*** | 0.952*** |
|  | [0.952,0.954] | [0.951,0.953] |

Note. * p<0.05, ** p<0.01, *** p<0.001. Abbreviations: ADHD = attention-deficit/hyperactivity disorder; OR = odds ratio; CI = confidence interval. Models adjusted for year, age, and birth month + parental education and migration background.

**Table G2. Associations between academic achievement and ADHD by parental education**

|  | Compulsory school | Upper secondary school | Tertiary education < 3 years | Tertiary education ≥ 3 years |
| --- | --- | --- | --- | --- |
|  | *OR[95% CI]* | *OR[95% CI]* | *OR[95% CI]* | *OR[95% CI]* |
| Raw grade total | 0.992*** | 0.987*** | 0.986*** | 0.986*** |
|  | [0.991,0.993] | [0.987,0.987] | [0.985,0.987] | [0.986,0.987] |
| Grade percentile | 0.957*** | 0.949*** | 0.953*** | 0.957*** |
|  | [0.952,0.962] | [0.947,0.950] | [0.951,0.955] | [0.956,0.958] |

Note. * p<0.05, ** p<0.01, *** p<0.001. Abbreviations: ADHD = attention-deficit/hyperactivity disorder; OR = odds ratio; CI = confidence interval. Models adjusted for year, age, and birth month + gender and migration background.

**Table G3. Associations between academic achievement and ADHD by migration background**

|  | Native | Second generation | First generation |
| --- | --- | --- | --- |
|  | *OR[95% CI]* | *OR[95% CI]* | *OR[95% CI]* |
| Raw grade total | 0.986*** | 0.989*** | 0.995*** |
|  | [0.986,0.986] | [0.988,0.990] | [0.994,0.995] |
| Grade percentile | 0.951*** | 0.959*** | 0.975*** |
|  | [0.951,0.952] | [0.955,0.962] | [0.972,0.978] |

Note. * p<0.05, ** p<0.01, *** p<0.001. Abbreviations: ADHD = attention-deficit/hyperactivity disorder; OR = odds ratio; CI = confidence interval. Models adjusted for year, age, and birth month + gender and parental education.

**Table G4. Logistic regression models with attention-deficit/hyperactivity disorder diagnosis as the outcome. Models stratified by achievement decile.**

|  | *Decile 1* | *Decile 2* | *Decile 3* | *Decile 4* | *Decile 5* | *Decile 6* | *Decile 7* | *Decile 8* | *Decile 9* | *Decile 10* |
| --- | --- | --- | --- | --- | --- | --- | --- | --- | --- | --- |
|  | *OR*  *[95% CI]* | *OR*  *[95% CI]* | *OR*  *[95% CI]* | *OR*  *[95% CI]* | *OR*  *[95% CI]* | *OR*  *[95% CI]* | *OR*  *[95% CI]* | *OR*  *[95% CI]* | *OR*  *[95% CI]* | *OR*  *[95% CI]* |
| Gender (ref: girl) |  |  |  |  |  |  |  |  |  |  |
| Boy | 1.34*** | 1.07 | 1.11* | 1.24*** | 1.20** | 1.08 | 1.48*** | 1.56*** | 1.53*** | 1.37 |
|  | [1.26,1.43] | [0.99,1.15] | [1.01,1.21] | [1.12,1.38] | [1.06,1.35] | [0.94,1.25] | [1.25,1.75] | [1.28,1.90] | [1.19,1.97] | [0.93,2.03] |
| Parental education (ref: compulsory) |  |  |  |  |  |  |  |  |  |  |
| Upper secondary | 1.58*** | 1.37*** | 1.23 | 1.31 | 1.82* | 1.17 | 2.26 | 2.03 | 2.93 | 1.08 |
|  | [1.41,1.77] | [1.18,1.61] | [0.99,1.54] | [0.96,1.79] | [1.13,2.94] | [0.71,1.94] | [0.91,5.65] | [0.63,6.55] | [0.40,21.53] | [0.64,1.82] |
| Tertiary < 3 years | 1.66*** | 1.65*** | 1.41** | 1.31 | 2.03** | 1.12 | 2.55* | 1.96 | 3.65 | 0.87 |
|  | [1.45,1.90] | [1.39,1.96] | [1.12,1.78] | [0.95,1.81] | [1.25,3.31] | [0.67,1.88] | [1.02,6.39] | [0.60,6.36] | [0.50,26.82] | [0.51,1.50] |
| Tertiary ≥ 3 years | 1.71*** | 1.85*** | 1.64*** | 1.55** | 2.82*** | 1.50 | 2.83* | 2.39 | 3.24 | - |
|  | [1.50,1.95] | [1.56,2.18] | [1.31,2.06] | [1.13,2.13] | [1.75,4.54] | [0.91,2.49] | [1.14,7.03] | [0.74,7.64] | [0.45,23.50] | - |
| Migration background (ref: native) |  |  |  |  |  |  |  |  |  |  |
| 2nd generation | 0.32*** | 0.41*** | 0.42*** | 0.58*** | 0.63*** | 0.53*** | 0.45*** | 0.56** | 0.63 | 0.65 |
|  | [0.28,0.36] | [0.36,0.47] | [0.35,0.51] | [0.48,0.71] | [0.50,0.80] | [0.39,0.72] | [0.30,0.68] | [0.36,0.87] | [0.36,1.10] | [0.29,1.50] |
| 1st generation | 0.08*** | 0.21*** | 0.29*** | 0.30*** | 0.46*** | 0.40*** | 0.63* | 0.54* | 0.96 | 0.67 |
|  | [0.07,0.10] | [0.18,0.25] | [0.24,0.35] | [0.24,0.38] | [0.35,0.59] | [0.29,0.55] | [0.45,0.90] | [0.34,0.87] | [0.59,1.56] | [0.28,1.59] |
| N | 27890 | 30346 | 31947 | 28857 | 29853 | 30325 | 30299 | 28961 | 30458 | 27238 |
|  |  |  |  |  |  |  |  |  |  |  |

Note. * p<0.05, ** p<0.01, *** p<0.001. Abbreviations: ADHD = attention-deficit/hyperactivity disorder; OR = odds ratio; CI = confidence interval. Models adjusted for year, age, and birth month + gender, parental education and migration background.
