# Supplementary material for: Academic achievement and socio-demographic differences in ADHD diagnosis: a population-based study of Swedish compulsory school pupils
Source: Child Adolesc Psychiatry Ment Health. 2026 Sep 9;20:133. doi: 10.1186/s13034-026-01176-4 (PMC13613661; doi:10.1186/s13034-026-01176-4)
Supplement: Supplementary file 1 [file 13034_2026_1176_MOESM1_ESM.zip › Additional file 9.docx]

**Additional file 9. Missing parental education data as separate category.**

In the main analysis, pupils with missing parental education data (n = 4,212) were excluded. Since missing parental education mostly concern immigrant parents whose foreign qualifications are not captured in Swedish registers, Additional file 9 retains these pupils as a separate category (total n = 300,592). The results are consistent with those of the main analysis across all sociodemographic dimensions. Pupils with missing parental education had markedly lower odds of ADHD diagnosis than any other group, both before (OR = 0.32) and after (OR = 0.34) adjustment for achievement, consistent with the small changes in the odds for immigrant pupils.

**Table H1. Logistic regression models with attention-deficit/hyperactivity disorder diagnosis as the outcome. Missing parental education as separate category.**

|  | *Column 1* | *Column 2* |
| --- | --- | --- |
|  | *OR[95% CI]* | *OR[95% CI]* |
| *Gender (ref: girl)* |  |  |
| Boy | 1.72*** | 1.20*** |
|  | [1.67,1.78] | [1.16,1.25] |
| *Parental education (ref: compulsory)* |  |  |
| Upper secondary | 1.13** | 1.58*** |
|  | [1.04,1.22] | [1.46,1.71] |
| Tertiary < 3 years | 0.75*** | 1.69*** |
|  | [0.69,0.82] | [1.55,1.85] |
| Tertiary ≥ 3 years | 0.61*** | 1.98*** |
|  | [0.56,0.66] | [1.82,2.15] |
| Missing | 0.32*** | 0.34*** |
|  | [0.25,0.42] | [0.26,0.44] |
| *Migration background (ref: native)* |  |  |
| 2nd generation | 0.47*** | 0.43*** |
|  | [0.44,0.50] | [0.40,0.46] |
| 1st generation | 0.24*** | 0.17*** |
|  | [0.23,0.26] | [0.16,0.18] |
| Grade percentile |  | 0.96*** |
|  |  | [0.96,0.96] |
| Grade sum |  | 0.99*** |
|  |  | [0.99,0.99] |
| Model | 2 | 3 |
| N | 300592 | 300592 |

Note. * p<0.05, ** p<0.01, *** p<0.001. Abbreviations: ADHD = attention-deficit/hyperactivity disorder; OR = odds ratio; CI = confidence interval. Model 2 (column 1): adjusted for year, age, and birth month + gender, parental education and migration background (simultaneously). Model 3 (column 2): adjusted for year, age, and birth month + gender, parental education and migration background + grade sum and percentile rank.
