# Supplementary material for: Academic achievement and socio-demographic differences in ADHD diagnosis: a population-based study of Swedish compulsory school pupils
Source: Child Adolesc Psychiatry Ment Health. 2026 Sep 9;20:133. doi: 10.1186/s13034-026-01176-4 (PMC13613661; doi:10.1186/s13034-026-01176-4)
Supplement: Supplementary file 1 [file 13034_2026_1176_MOESM1_ESM.zip › Additional file 10.docx]

**Additional file 10. Robustness to diagnostic timing.**

Additional file 10 examines whether differences in the timing of first ADHD diagnosis affect the results. Table I1 adjusts for ADHD diagnosis (specialised care visits or medications) recorded in years t-5 to t-2, with t = year of graduation. Table 12 restricts the outcome to pupils with a first recorded diagnosis or medication in years t-1 or t, excluding those with a diagnosis in years t-5 to t-2. Only data from years t to t-5 were available.

Results for SES and migration background are similar to the main results in both tables. The gender results differ, however. When earlier diagnoses are accounted for, the male excess in Model 2 is substantially reduced, and after adjustment for achievement (Model 3) girls have higher odds of diagnosis than boys. This is consistent with the well-documented pattern of later diagnosis in girls, with Swedish girls having a higher incidence than boys from about age 15 (National Board of Health and Welfare, 2023).

*Reference*

National Board of Health and Welfare. Diagnostik och läkemedelsbehandling vid adhd: Förekomst, trend och könsskillnader. Report no. 2023-11-8862. Stockholm: National Board of Health and Welfare; 2023.

**Table I1. Logistic regression models with attention-deficit/hyperactivity disorder diagnosis as the outcome. Adjusted for earlier ADHD diagnosis.**

|  | *Column 1* | *Column 2* |
| --- | --- | --- |
|  | *OR[95% CI]* | *OR[95% CI]* |
| *Gender (ref: girl)* |  |  |
| Boy | 1.23*** | 0.89*** |
|  | [1.18,1.28] | [0.85,0.93] |
| *Parental education (ref: compulsory)* |  |  |
| Upper secondary | 1.16** | 1.65*** |
|  | [1.06,1.28] | [1.50,1.82] |
| Tertiary < 3 years | 0.80*** | 1.77*** |
|  | [0.72,0.89] | [1.60,1.97] |
| Tertiary ≥ 3 years | 0.69*** | 2.14*** |
|  | [0.62,0.76] | [1.93,2.37] |
| *Migration background (ref: native)* |  |  |
| 2nd generation | 0.51*** | 0.48*** |
|  | [0.47,0.56] | [0.44,0.52] |
| 1st generation | 0.37*** | 0.24*** |
|  | [0.34,0.40] | [0.22,0.26] |
| Grade percentile |  | 0.96*** |
|  |  | [0.96,0.96] |
| Grade sum |  | 0.99*** |
|  |  | [0.99,0.99] |
| Model | 2 | 3 |
| N | 296380 | 296380 |

Note. * p<0.05, ** p<0.01, *** p<0.001. Abbreviations: ADHD = attention-deficit/hyperactivity disorder; OR = odds ratio; CI = confidence interval. Model 2 (column 1): adjusted for year, age, and birth month + gender, parental education, migration background, and ADHD diagnosis in years t-5 to t-2. Model 3 (column 2): adjusted for year, age, and birth month + gender, parental education and migration background + grade sum and percentile rank.

**Table I2. Logistic regression models with attention-deficit/hyperactivity disorder diagnosis as the outcome. ADHD measured as new diagnoses in year t-1 or t.**

|  | *Column 1* | *Column 2* |
| --- | --- | --- |
|  | *OR[95% CI]* | *OR[95% CI]* |
| *Gender (ref: girl)* |  |  |
| Boy | 1.22*** | 0.84*** |
|  | [1.17,1.27] | [0.80,0.87] |
| *Parental education (ref: compulsory)* |  |  |
| Upper secondary | 1.11* | 1.58*** |
|  | [1.00,1.23] | [1.42,1.75] |
| Tertiary < 3 years | 0.75*** | 1.71*** |
|  | [0.67,0.84] | [1.53,1.91] |
| Tertiary ≥ 3 years | 0.65*** | 2.14*** |
|  | [0.58,0.72] | [1.92,2.38] |
| *Migration background (ref: native)* |  |  |
| 2nd generation | 0.53*** | 0.49*** |
|  | [0.48,0.57] | [0.45,0.54] |
| 1st generation | 0.32*** | 0.22*** |
|  | [0.29,0.35] | [0.20,0.24] |
| Grade percentile |  | 0.96*** |
|  |  | [0.96,0.96] |
| Grade sum |  | 0.99*** |
|  |  | [0.99,0.99] |
| Model | 2 | 3 |
| N | 296380 | 296380 |

Note. * p<0.05, ** p<0.01, *** p<0.001. Abbreviations: ADHD = attention-deficit/hyperactivity disorder; OR = odds ratio; CI = confidence interval. Model 2 (column 1): adjusted for year, age, and birth month + gender, parental education and migration background (simultaneously). Model 3 (column 2): adjusted for year, age, and birth month + gender, parental education and migration background + grade sum and percentile rank.
