# Supplementary material for: Academic achievement and socio-demographic differences in ADHD diagnosis: a population-based study of Swedish compulsory school pupils
Source: Child Adolesc Psychiatry Ment Health. 2026 Sep 9;20:133. doi: 10.1186/s13034-026-01176-4 (PMC13613661; doi:10.1186/s13034-026-01176-4)
Supplement: Supplementary file 1 [file 13034_2026_1176_MOESM1_ESM.zip › Additional file 1.docx]

**Additional file 1. Directed acyclic graph**

Arrows represent the main hypothesised pathways. Sociodemographic characteristics can shape the probability of ADHD diagnosis through three pathways:

1. By influencing the probability of the underlying condition itself (or ADHD symptoms).
2. By influencing academic achievement, either directly or indirectly through ADHD symptoms, which in turn influences the probability of diagnosis.
3. Independently of both ADHD symptoms and achievement (a direct effect)

In the regression models, academic achievement is included as a covariate in model 3. In DAG terms, conditioning on achievement blocks all pathways that run through it: specifically, the path ADHD → Achievement → Diagnosis and the path Sociodemographic → Achievement → Diagnosis.

Two pathways from sociodemographic characteristics to diagnosis therefore remain open after adjustment:

- The path running through ADHD (Sociodemographic → ADHD → Diagnosis). This reflects genuine differences in the prevalence or severity of underlying ADHD symptoms across groups net of achievement. Achievement serves as an imperfect proxy for ADHD symptoms, and this path can be seen as the residual association that capture ADHD symptoms that do not interfere with the more narrow form of academic functioning captured by compulsory school grades.
- The direct path (Sociodemographic → Diagnosis). This path captures differential access to, or recognition of, diagnosis independent of symptom burden, for instance SES differences in parental help-seeking or differential clinical recognition across groups.

The adjusted estimates in Model 3 reflect the combined contribution of these two remaining pathways.
