# Supplementary material for: Academic achievement and socio-demographic differences in ADHD diagnosis: a population-based study of Swedish compulsory school pupils
Source: Child Adolesc Psychiatry Ment Health. 2026 Sep 9;20:133. doi: 10.1186/s13034-026-01176-4 (PMC13613661; doi:10.1186/s13034-026-01176-4)
Supplement: Supplementary file 1 [file 13034_2026_1176_MOESM1_ESM.zip › Additional file 2.docx]

**Additional file 2. Summary statistics on socio-demographic characteristics and academic achievement**

**Table A1. Socio-demographic differences in academic achievement**

|  | *Mean raw grade total* | *Mean percentile rank* |
| --- | --- | --- |
| *Gender* |  |  |
| Girl | 230.2 | 56.7 |
| Boy | 207.0 | 44.3 |
| *Parenal education* |  |  |
| Compulsory | 150.0 | 25.6 |
| Upper secondary | 200.4 | 41.1 |
| Tertiary < 3 years | 225.0 | 52.8 |
| Tertiary ≥ 3 years | 245.2 | 63.0 |
| *Migration background* |  |  |
| Native | 225.2 | 52.9 |
| 2nd generation | 214.9 | 48.2 |
| 1st generation | 178.7 | 36.6 |

**Table A2. Parental education by migration background.**

|  | Compulsory | Upper secondary | Tertiary < 3 years | Tertiary ≥ 3 years |
| --- | --- | --- | --- | --- |
| Native | 1.89 % | 40.04 % | 18.94 % | 39.13 % |
| 2nd generation | 11.97 % | 45.34 % | 16.07 % | 26.63 % |
| 1st generation | 26.91 % | 27.78 % | 16.25 % | 29.06 % |
