# Supplementary material for: Academic achievement and socio-demographic differences in ADHD diagnosis: a population-based study of Swedish compulsory school pupils
Source: Child Adolesc Psychiatry Ment Health. 2026 Sep 9;20:133. doi: 10.1186/s13034-026-01176-4 (PMC13613661; doi:10.1186/s13034-026-01176-4)
Supplement: Supplementary file 1 [file 13034_2026_1176_MOESM1_ESM.zip › Additional file 3.docx]

**Additional file 3. Exact coefficients for Figures 1-3**

**Table B1. Predicted probability of attention-deficit/hyperactivity disorder diagnosis**

|  | *Model 2* | *Model 3* |
| --- | --- | --- |
|  | *Predicted probability*  *[95% CI]* | *Predicted probability*  *[95% CI]* |
| *Gender* |  |  |
| Girl | 0.042*** | 0.051*** |
|  | [0.041,0.043] | [0.050,0.052] |
| Boy | 0.069*** | 0.060*** |
|  | [0.068,0.071] | [0.059,0.061] |
| *Parenal education* |  |  |
| Compulsory | 0.064*** | 0.036*** |
|  | [0.059,0.068] | [0.034,0.038] |
| Upper secondary | 0.072*** | 0.054*** |
|  | [0.071,0.074] | [0.053,0.056] |
| Tertiary < 3 years | 0.050*** | 0.058*** |
|  | [0.048,0.052] | [0.056,0.060] |
| Tertiary ≥ 3 years | 0.041*** | 0.066*** |
|  | [0.040,0.042] | [0.064,0.068] |
| *Migration background* |  |  |
| Native | 0.068*** | 0.072*** |
|  | [0.067,0.069] | [0.071,0.073] |
| 2nd generation | 0.034*** | 0.035*** |
|  | [0.032,0.036] | [0.033,0.037] |
| 1st generation | 0.018*** | 0.015*** |
|  | [0.017,0.020] | [0.014,0.016] |
| N | 296380 | 296380 |

Note. Note. * p<0.05, ** p<0.01, *** p<0.001. Abbreviations: CI = confidence interval. Table presents predicted probabilities of ADHD diagnosis from logistic regression models. Model 2: adjusted for year, age, and birth month. Model 3: adjusted for year, age, birth month, and raw grade total and percentile rank. Predicted probabilities represent the average predicted probability for each group, holding the distribution of all other covariates constant.
