# Supplementary material for: Academic achievement and socio-demographic differences in ADHD diagnosis: a population-based study of Swedish compulsory school pupils
Source: Child Adolesc Psychiatry Ment Health. 2026 Sep 9;20:133. doi: 10.1186/s13034-026-01176-4 (PMC13613661; doi:10.1186/s13034-026-01176-4)
Supplement: Supplementary file 1 [file 13034_2026_1176_MOESM1_ESM.zip › Additional file 4.docx]

**Additional file 4. Only data from the National Patient Register used to define ADHD**

Additional file 4 shows results when only data from the National Patient Register (ICD-10: F90), not data from the Prescribed Drug Register, was used to measure ADHD. This is motivated by socio-demographic differences in medication use, given ADHD diagnosis (Kok et al., 2020; Pearce et al., 2024). Note that diagnosis is measured as any specialised inpatient or outpatient care visit with an F90 code in the year before or the same year that the pupil graduated from compulsory school.

Table C1 shows that 88% (11602/13171) of pupils with a diagnosis in the relevant time period also received ADHD medication during this time period, while 77% (11602/15080) of pupils with ADHD medication in the relevant time period also received a diagnosis in this time period.

Table C2 shows that the key results concerning changes in the socio-demographic differences are very similar when only data on diagnosis are used.

*References*

Kok, F. M., Groen, Y., Fuermaier, A. B. M., & Tucha, O. (2020). The female side of pharmacotherapy for ADHD—A systematic literature review. *PLOS ONE*, *15*(9), e0239257. <https://doi.org/10.1371/journal.pone.0239257>

Pearce, A., Henery, P., Katikireddi, S. V., Dundas, R., Leyland, A. H., Nicholls, D., Viner, R. M., Fenton, L., & Hope, S. (2024). Childhood attention-deficit hyperactivity disorder: socioeconomic inequalities in symptoms, impact, diagnosis and medication. *Child and Adolescent Mental Health*, *29*(2), 126-135. <https://doi.org/https://doi.org/10.1111/camh.12707>

**Table C1. Number of pupils with ADHD diagnosis or pharmacological treatment.**

|  | ADHD medication: No | ADHD medication: Yes | Total |
| --- | --- | --- | --- |
| Diagnosis: No | 279731 | 3478 | 283209 |
| Diagnosis: Yes | 1569 | 11602 | 13171 |
| Total | 281300 | 15080 |  |

**Table C2. Logistic regression models with attention-deficit/hyperactivity disorder diagnosis as the outcome. Only data from the National Patient Register used to define ADHD**

|  | *Model 2* | *Model 3* |
| --- | --- | --- |
|  | *OR[95% CI]* | *OR[95% CI]* |
| *Gender (ref: girl)* |  |  |
| Boy | 1.71*** | 1.20*** |
|  | [1.65,1.78] | [1.15,1.25] |
| *Parental education (ref: compulsory)* |  |  |
| Upper secondary | 1.12** | 1.54*** |
|  | [1.03,1.22] | [1.41,1.68] |
| Tertiary < 3 years | 0.75*** | 1.63*** |
|  | [0.68,0.82] | [1.48,1.79] |
| Tertiary ≥ 3 years | 0.59*** | 1.84*** |
|  | [0.54,0.65] | [1.68,2.02] |
| *Migration background (ref: native)* |  |  |
| 2nd generation | 0.50*** | 0.47*** |
|  | [0.47,0.54] | [0.44,0.51] |
| 1st generation | 0.26*** | 0.19*** |
|  | [0.24,0.29] | [0.18,0.21] |
| Grade percentile |  | 0.96*** |
|  |  | [0.96,0.96] |
| Raw grade total |  | 0.99*** |
|  |  | [0.99,0.99] |
| N | 296380 | 296380 |

Note. * p<0.05, ** p<0.01, *** p<0.001. Abbreviations: ADHD = attention-deficit/hyperactivity disorder; OR = odds ratio; CI = confidence interval. Model 2: adjusted for year, age, and birth month + gender, parental education and migration background (simultaneously). Model 3: adjusted for year, age, and birth month + gender, parental education and migration background + raw grade total and percentile rank.
