# Supplementary material for: Academic achievement and socio-demographic differences in ADHD diagnosis: a population-based study of Swedish compulsory school pupils
Source: Child Adolesc Psychiatry Ment Health. 2026 Sep 9;20:133. doi: 10.1186/s13034-026-01176-4 (PMC13613661; doi:10.1186/s13034-026-01176-4)
Supplement: Supplementary file 1 [file 13034_2026_1176_MOESM1_ESM.zip › Additional file 5.docx]

**Additional file 5. Diagnoses measured up to five years prior to graduation**

Additional file 5 shows results when ADHD is measured as any specialised inpatient or outpatient care visit with an F90 code in the five years prior to, or the same year as, compulsory school graduation. Only data from the National Patient Register were available for these analyses. Results show that the key results concerning changes in the socio-demographic differences are very similar.

**Table D1. Logistic regression models with attention-deficit/hyperactivity disorder diagnosis as the outcome. Diagnoses measured up to five years prior to graduation**

|  | *Model 2* | *Model 3* |
| --- | --- | --- |
|  | *OR[95% CI]* | *OR[95% CI]* |
| *Gender (ref: girl)* |  |  |
| Boy | 1.87*** | 1.31*** |
|  | [1.81,1.94] | [1.27,1.36] |
| *Parental education (ref: compulsory)* |  |  |
| Upper secondary | 1.10* | 1.52*** |
|  | [1.01,1.19] | [1.40,1.65] |
| Tertiary < 3 years | 0.73*** | 1.62*** |
|  | [0.67,0.80] | [1.48,1.77] |
| Tertiary ≥ 3 years | 0.59*** | 1.86*** |
|  | [0.54,0.64] | [1.71,2.03] |
| *Migration background (ref: native)* |  |  |
| 2nd generation | 0.52*** | 0.49*** |
|  | [0.49,0.56] | [0.46,0.52] |
| 1st generation | 0.27*** | 0.19*** |
|  | [0.25,0.29] | [0.18,0.21] |
| Grade percentile |  | 0.96*** |
|  |  | [0.96,0.96] |
| Raw grade total |  | 0.99*** |
|  |  | [0.99,0.99] |
| N | 296380 | 296380 |

Note. * p<0.05, ** p<0.01, *** p<0.001. Abbreviations: ADHD = attention-deficit/hyperactivity disorder; OR = odds ratio; CI = confidence interval. Model 2: adjusted for year, age, and birth month + gender, parental education and migration background (simultaneously). Model 3: adjusted for year, age, and birth month + gender, parental education and migration background + raw grade total and percentile rank.
